# Bud development and shoot morphology in relation to crown location

**DOI:** 10.1093/aobpla/plv082

**Published:** 2015-07-17

**Authors:** Maarja Kukk, Anu Sõber

**Affiliations:** Institute of Ecology and Earth Sciences, University of Tartu, Lai 40, 51005 Tartu, Estonia

**Keywords:** Allometry, architecture, bud mass, current-year shoot, light, neoformation, preformation, vertical crown gradient

## Abstract

Plant architecture is shaped by endogenous growth processes interacting with the local environment. The current study investigated crown development in young black alder trees, assessing the effects of local light conditions and branch height on individual bud mass and contents. In addition, we examined the characteristics of parent shoots [the cross-sectional area (CSA) of stem and total leaf area, shoot length, the number of nodes, the number and total mass of buds per shoot] and leaf–stem as well as bud–stem allometry, as several recent studies link bud development to hydraulic architecture. We sampled shoots from top branches and two lower-crown locations: one subjected to deep shade and the other resembling the upper branches in light availability. Sampling was carried out three times between mid-July and late October, spanning from the early stages of bud growth to dormancy. Individual bud mass and shoot characteristics varied in response to light conditions, whereas leaf–stem allometry depended on branch height, most likely compensating for the increasing length of hydraulic pathways. Despite the differences in individual bud mass, the number of preformed leaves varied little across the crown, indicating that the plasticity in shoot characteristics was mainly achieved by neoformation. The relationship between total bud mass and stem CSA scaled similarly across crown locations. However, scaling slopes gradually decreased throughout the sampling period, driven by bud rather than by stem growth. This suggests that the allometry of total bud mass and CSA of stem is regulated locally, instead of resulting from crown-level processes.

## Abstract (precis)

We investigated crown development in young trees, assessing whether the plasticity in parent shoot and bud growth is mostly related to the position within a crown (upper versus lower branches) or driven by local light conditions. Both larger shoots and buds were associated with increased light availability, whereas leaf-stem allometry was influenced by branch height. The allometry of total bud mass and stem cross-sectional area varied little across the crown, but it changed throughout the summer, driven by bud rather than stem growth. This suggests that bud-stem relationships are regulated locally, at the shoot level, instead of crown-level processes.

## Introduction

Tree crowns develop by forming semi-autonomous repeated elements: buds, metamers, shoots and branches (White 1979; Maillette 1982*a*, *b*; Barthélémy and Caraglio 2007; Kawamura 2010). In young, fast-growing trees, bud production is prolific at the top of the crown, whereas lower branches degrade, as buds are fewer, prone to higher mortality rates and tend to produce short, low-vigour shoots adjusted to leaf display rather than crown expansion (Maillette 1982*a*; Jones and Harper 1987*a*, *b*; Kimura *et al*. 1998; Kull and Tulva 2002; Remphrey *et al*. 2002). Unequal rates of bud production promote a rapid gain in height over crown width (Maillette 1982*b*), allowing young trees to escape from unfavourable, shaded environments (Henry and Aarssen 2001).

Plant architecture is shaped by endogenous processes that interact with the local environment (Barthélémy and Caraglio 2007; Kawamura 2010). Local conditions vary widely within the crown of a tree. Most obviously, the upper and outer parts of a crown receive more light, but suffer also from greater heat and moisture stress and stronger winds than the lower branches or the interior of a crown (Niinemets and Valladares 2004). Light is a crucial factor affecting bud characteristics: buds formed in shade are smaller, exhibit lower probabilities of bud break in the following spring (Sanz-Pérez and Castro-Díez 2010) and produce shoots consisting of fewer nodes (Kimura *et al*. 1998). Besides organ initiation, light availability also influences the anatomical development of incipient leaves (Eschrich *et al*. 1989; Uemura *et al*. 2000). Accordingly, contrasting light conditions contribute to determining the differing rates of bud production, mortality and the characteristics of the future shoots in the upper versus lower crown (Jones and Harper 1987*a*, *b*; Eschrich *et al*. 1989; Kimura *et al*. 1998; Uemura *et al*. 2000; Kull and Tulva 2002).

However, differences in bud characteristics are also frequently attributed to the position within the architecture of a plant (e.g. Barthélémy and Caraglio 2007). Along a parent shoot, buds positioned distally are larger, contain a greater number of preformed organs and display higher probabilities of bud break than basal buds (Gill 1971; Remphrey and Davidson 1994; Sabatier and Barthélémy 2001; Gordon *et al*. 2006; Alla *et al*. 2013*b*). The mechanisms implicated in determining bud characteristics include hormonal signalling (Cline 2000; Djennane *et al*. 2014) and resource competition among buds (Cline *et al*. 2009; Mason *et al*. 2014), but several recent studies also highlight the importance of vascular connections between buds and stem. The number of leaf primordia found in overwintering buds is correlated to xylem area at the base of the parent shoot as well as to the hydraulic conductance of the vascular pathway leading to the buds (Cochard *et al*. 2005). Furthermore, the vascular differentiation of embryonic shoots is a key step in the sequence of events leading to bud break in spring (de Faÿ *et al*. 2000; Sutinen *et al*. 2012), and buds failing to establish a vascular connection to the stem remain dormant or abort (Han *et al*. 2007; Lauri *et al*. 2008). Unlike current-year shoots, the contribution of these endogenous mechanisms is less clear at the crown level, as potential topological effects are confounded by environmental heterogeneity.

Developmental and functional interdependencies among shoot components lead to coordinated growth in response to prevailing conditions. Due to both mechanical and hydraulic reasons, the leaf area supported by a shoot depends on the cross-sectional area (CSA) of stem (Preston and Ackerly 2003; Normand *et al*. 2008). Consequently, leaf–stem allometry responds to environmental gradients across habitats (Preston and Ackerly 2003; Westoby and Wright 2003; Sun *et al*. 2006) and seasonal changes in precipitation (Bucci *et al*. 2005). The leaf area supported by a given stem area also decreases as trees grow taller, to overcome the hydraulic stress imposed by the increasing length of resource transport pathways and gravity (McDowell *et al*. 2002). In contrast, studies investigating the allometry of current-year shoots rarely involve buds. Alla *et al*. (2011) have examined the scaling of apical bud mass and stem CSA, concluding that bud–stem relationships are influenced by environmental factors, but bud–stem allometry along the vertical crown gradient and especially for axillary buds remains unexplored.

The current study investigated crown development in young black alder trees. We examined individual bud mass and contents, the characteristics of current-year shoots, and leaf–stem and bud–stem allometry. Sampling was carried out in three crown locations to assess the effects of two underlying factors: light availability and branch height. The characteristics of parent shoots were expected to respond mainly to light conditions, and improved light availability was also expected to increase individual bud mass and the number of leaf primordia per bud. However, due to the potential link between bud development and hydraulic architecture, we hypothesized that similarly to leaf–stem allometry, the relationship between total bud mass and stem CSA would mainly respond to branch height. To gain further insight, we also examined the allometry of individual bud mass and the CSA of stem. Sampling was carried out three times between mid-July and late October, to investigate whether the effects of crown location on individual bud mass and on bud–stem allometry vary throughout the period of rapid bud development.

## Methods

### Study system

The current study was conducted in a small stand of black alder (*Alnus glutinosa* (L.) Gaertn.) trees, located on former agricultural land in Rõka village, south-eastern Estonia (58°15′N, 27°18′E; 50 m above sea level). The soil in the study area is Endogleyic Planosol (Hansen *et al*. 2013). Long-term mean temperature is +16.9 °C in July and −5.4 °C in February; mean annual precipitation amounts to 637 mm. Black alder is a light-demanding deciduous tree species, preferring wet habitats. The stand consists of even-aged trees, planted in a 2 × 2 m grid in 2007. By the time of sampling in 2013, trees had reached the height of 6–7 m and formed a closed canopy.

### Sampling

Sampling was carried out on five trees growing at the edge of the stand. The edge follows NW–SE direction, facing an open field to the west of the stand. On average, sample trees measured 7.1 ± 0.7 m in height (±SD) and had the diameter of 8.2 ± 1.7 cm at 1.3 m from ground level.

Current-year shoots were collected from three crown locations: lower-crown branches facing the interior or the edge of the stand (LI and LE, respectively), and upper-crown (U) branches facing the edge. Sampling was carried out three times. Shoots were first collected during the early stages of bud development (July); further sampling was carried out near the end of the growing season (August) and finally, dormant shoots were collected after leaf drop (October). At any given sampling date, one lateral branch per crown location was selected from each sample tree. Selected branches were 5 year olds in the lower crown and 2–3 year olds in the upper crown, issuing from the trunk at the height of ∼1.5 m (LI and LE branches) and 4 m (U branches), respectively (Table 1).

**Table 1.** The heights of sample branches (mean ± SD, *n* = 5), sample sizes (the total number of shoots sampled per crown location with the number of shoots per branch in brackets) and the percentage of light transmitted through the canopy (TSF, total site factor; mean ± SD, *n* = 20). LI, lower-crown, interior-facing branches; LE, lower-crown, edge-facing branches; U, upper crown.

Sampling date	Crown location		
	LI	LE	U
Branch height			
11 July 2013	152 ± 27 cm	141 ± 26 cm	381 ± 37 cm
25 August 2013	182 ± 27 cm	170 ± 26 cm	423 ± 38 cm
25 October 2013	138 ± 62 cm	137 ± 62 cm	361 ± 36 cm
Sample size			
11 July 2013	36 (5–10)	49 (9–10)	48 (9–10)
25 August 2013	35 (5–8)	46 (8–10)	43 (7–10)
25 October 2013	40 (8)	40 (8)	40 (8)
TSF			
13 July 2014	12.4 ± 2.9 %	32.8 ± 17.9 %	47.1 ± 16.6 %

In July and August, 10 randomly selected shoots were harvested per branch. However, a few of these shoots were later found to carry mainly dead buds (discernible by ready abscission) and were discarded, resulting in slightly reduced sample sizes (Table 1). In October, eight shoots, each carrying viable buds, were sampled per branch. Harvested shoots were stored in sealed plastic bags at +5 °C for 2–5 days until further measurements.

The sampled crown locations were exposed to differing light environments. Light availability was quantified using hemispherical photography in the following summer. Four photographs per location, covering both the periphery and the interior of the crown, were taken from each sample tree, using a horizontally levelled digital camera (Coolpix 950, Nikon) equipped with a fisheye lens (LC-ER1, Nikon). Photographs were taken above branches located ∼0.5 m higher than the branches sampled in the previous summer. As trees had gained height, new branches corresponded to the same relative position and degree of shading within the canopy as the previous summer sample branches. Photographs were analysed using the WinScanopy software (version 2001a, Regent Instruments Inc.) to estimate the percentage of light transmitted through the canopy (i.e. total site factor). On average, U branches received nearly four times the light of LI branches, but only ∼40 % more light than LE branches (Table 1). Thus, the sampling design included a steep (U versus LI) as well as a shallow (U versus LE) vertical light gradient. By comparing the three crown locations, we were therefore able to assess the effects of two underlying factors: similar values for LI and LE branches but different for U branches were attributed to the difference in branch height, whereas similar values for LE and U branches but different for LI branches were presumably related to light availability.

### Measured variables

The characteristics determined for sampled shoots included the CSA of stem, shoot length, the number of nodes, leaf area, the number of buds and total bud mass per shoot. In addition, a bud was sampled from each shoot to assess individual bud mass and contents (i.e. the number of leaf primordia per bud).

The CSA of stem was calculated based on stem diameter, assuming that the cross-section is circular. Diameter was measured with a digital calliper at shoot base (the mean of two perpendicular measurements, precision 0.01 mm). Shoot length was measured with a ruler (precision 1 mm). In July and August, the leaf area per shoot was obtained by summing the areas of individual leaves. Leaf areas were predicted based on leaf length × width, using a linear regression (log_e_(area) = −5.31 + 1.04 × log_e_(length × width), *R*^2^ = 99.3 %, *P* < 0.001, *n* = 60). During field sampling in August, leaves for the model were randomly selected from each crown location, but the data were pooled, since leaf shape was similar across the crown (data not shown). The lengths and widths of leaf blades were determined using a ruler (precision 1 mm), and the areas were measured to the nearest 0.01 cm^2^ using an optical area meter (LI-3100C, LI-COR, Inc., Lincoln, NE, USA).

Buds were then carefully cut from the stems below the first bud scale. In July, buds were dried at 60 °C for 48 h and weighed to determine total bud mass per shoot (precision 0.1 mg); subsequently, a bud was selected randomly from each shoot and weighed to assess individual bud mass. The number of leaf primordia was not determined in July, because buds were still small and their contents could not be reliably identified. In August and October, sample buds were set aside before drying, weighed and dissected under a stereomicroscope (×16–40 magnification) to count the number of true leaf primordia. In black alder, each leaf primordium is flanked by two stipules, and the outer stipules function as bud scales; consequently, the number of true leaf primordia and the total number of organs are highly correlated. The dry mass of dissected buds (i.e. individual bud mass) was predicted based on fresh mass, using linear regressions fitted on buds that were additionally collected during field sampling (*R*^2^ = 96.1–98.8 %, *P* < 0.001, *n* = 16–23 for each combination of location and sampling date; data not shown). Thus, in August and October, total bud mass per shoot was obtained by adding the dry mass of the sample bud to the rest of the buds on a given shoot.

### Statistical analysis

Analyses were carried out using the statistics software R (R Development Core Team 2014).

The effects of crown location and sampling month on shoot and bud characteristics were analysed using mixed-effects modelling: random effects included sample tree and branch (nested within tree). Continuous variables were tested by mixed-effects analysis of variance, using package *nlme* (Pinheiro *et al*. 2014), and counts were tested using mixed-effects Poisson regression implemented in package *lme4* (Bates *et al*. 2014). When necessary, continuous variables were transformed, and non-significant interactions between the fixed effects were dropped. *P*-values for the fixed effects were based on type III sums of squares and were obtained using conditional *F*-tests (ANOVAs) or Wald χ^2^ tests (Poisson regressions), the latter of which were carried out using package *car* (Fox and Weisberg 2011).

Tests for the effects of crown location and sampling month were followed by pair-wise comparisons of group means. To this end, new one-way mixed-effects models were fitted for each dependent variable, containing the combination of crown location and sampling month as the fixed effect. Pair-wise comparisons were then carried out using simultaneous inference procedures implemented in package *multcomp* (Hothorn *et al*. 2008). Comparisons were set up among crown locations within each month and among sampling months within each location, as other possible pair-wise comparisons conveyed little useful meaning. Differences were deemed significant at *P* < 0.05.

Leaf area and stem CSA, total bud mass and stem CSA, and individual bud mass and stem CSA are related allometrically, approximating to a power law: *y* = *a* × *x^b^*. The relationships between shoot components were therefore linearized using log_10_(*y*) − log_10_(*x*) transformation. Linear mixed-effects models fitted for log_10_(leaf area), log_10_(total bud mass) and log_10_(individual bud mass) indicated that the variance not captured by fixed effects (log_10_(stem CSA), crown location and sampling month) was mainly accounted for by individual shoots (i.e. residual variance). The random effects of sample tree and branch contributed only 18.5, 11.9 and 18.2 % depending on the model (data not shown). Therefore, we decided to drop tree and branch effects when analysing shoot allometry, and instead pooled the data within each combination of crown location and sampling month.

Log–log-transformation puts variables on a multiplicative scale, and consequently, the slope of a bivariate relationship characterizes the scaling of the two variables relative to one another (i.e. slope = 1 means that if *x* is doubled, *y* is doubled as well, slope = 2 means that if *x* is the doubled, *y* is quadrupled, etc.). The relationships were fitted in each group (i.e. each combination of location and month) using standardized major axis (SMA) estimation. We relied on pair-wise comparisons to investigate whether the allometries scale differently across crown locations or sampling months. Using a test based on the likelihood ratio (LR) statistic, groups were first tested for a common slope, followed by pair-wise comparisons, if the global test was deemed significant. Again, comparisons were carried out among crown locations within each month and among sampling months within each location. The *P*-values of pair-wise comparisons were adjusted using Bonferroni correction, and differences were deemed significant at *P* < 0.05. If slopes were homogeneous, differences in *y*-intercepts were investigated, using a test based on the Wald statistic (W). Similarly to slopes, a global test was carried out, followed by pair-wise comparisons. Standardized major axis estimation and tests for slopes and intercepts were performed using the functions implemented in package *smatr* (Warton *et al*. 2012); the underlying methodology is described in more detail by Warton *et al*. (2006). Pearson's correlations coefficients between the leaf area and stem CSA or bud mass and stem CSA were calculated based on log_10_-transformed variables.

## Results

### Individual bud mass and contents

**Figure 1.**
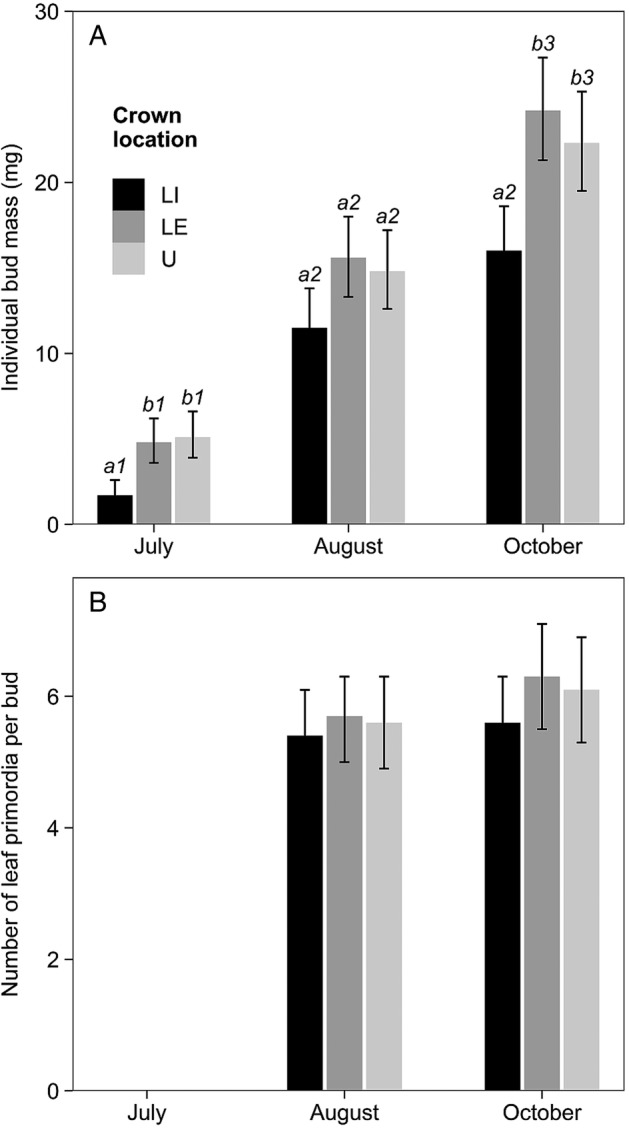
Mean individual bud mass (A) and mean number of leaf primordia per bud (B) in relation to crown location and sampling month. Error bars denote 95 % CI. Different letters indicate significant differences among crown locations (within each month), and different numbers indicate significant differences across sampling months (within each location). LI, lower-crown, interior-facing branches; LE, lower-crown, edge-facing branches; U, upper crown.

Individual bud mass depended significantly on crown location and sampling month (Table 2). On average, LI buds had lower mass than both LE and U buds, and pair-wise comparisons revealed that the differences were significant in July and October, although not in August (Fig. 1A). In contrast, pair-wise comparisons showed that mean individual bud mass was similar in LE and U branches throughout the study. Between July and August, mean individual bud mass increased significantly in all three locations, and the further mass increase between August and October was significant in LE and U branches. Despite the differences in individual bud mass, the average number of leaf primordia was unaffected by crown location, and sample buds contained a similar number of leaf primordia in August and October (Table 2, Fig. 1B).

**Table 2.** The effects of crown location and sampling month on individual bud mass and contents. Individual bud mass (square-root-transformed) was analysed using mixed-effects ANOVA and the number of leaf primordia by mixed-effects Poisson regression (nested random effects: sample tree and branch).

Variable	Fixed effect			
	Crown location		Sampling month	
Individual bud mass	*F*(2, 36) = 24.4	*P* < 0.001	*F*(2, 36) = 239.7	*P* < 0.001
Number of primordia	χ^2^(2) = 1.8	*P* = 0.4	χ^2^(1) = 2.5	*P* = 0.1

### Shoot characteristics

**Figure 2.**
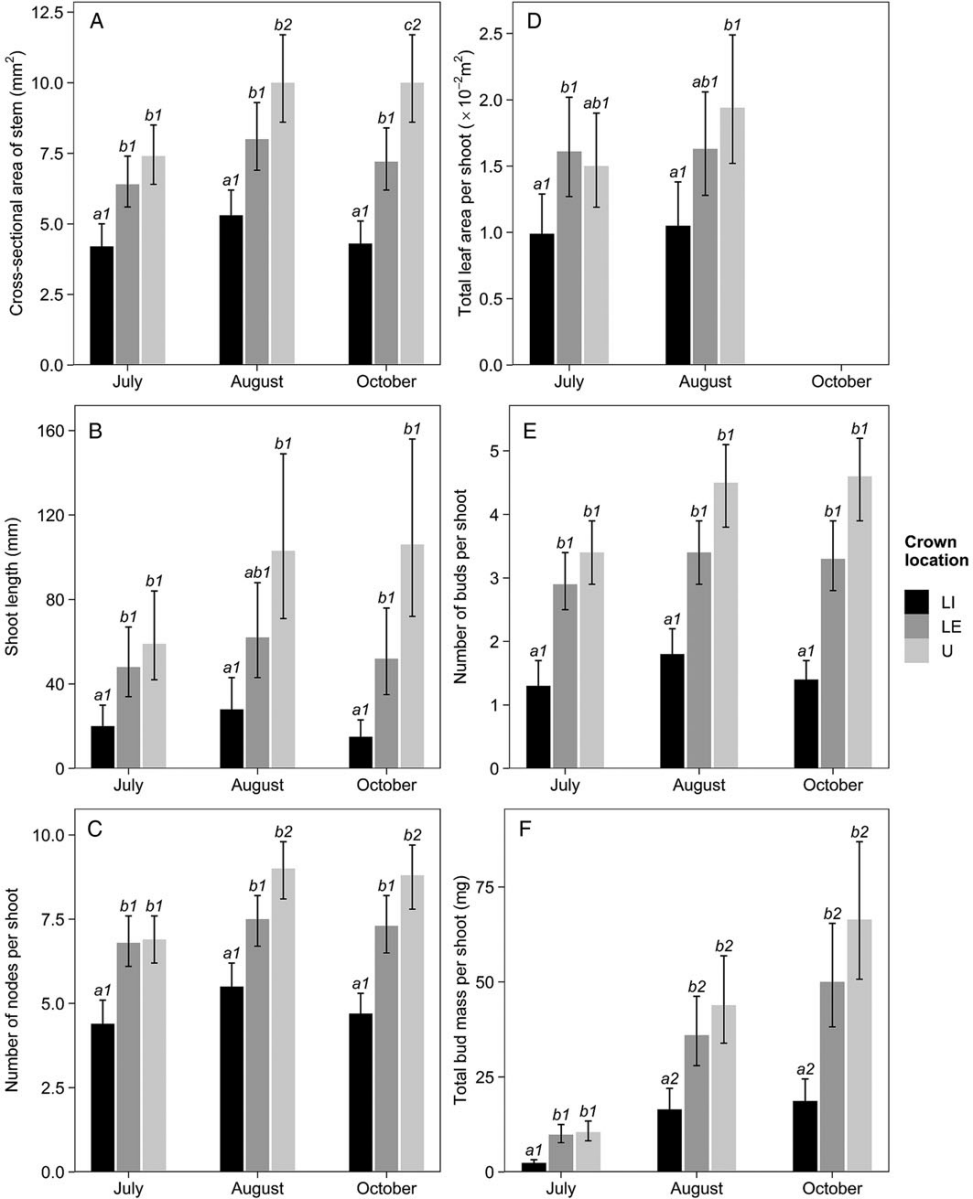
Mean values of shoot characteristics in relation to crown location and sampling month: CSA of stem (A), shoot length (B), number of nodes per shoot (C), total leaf area per shoot (D), number of buds per shoot (E) and total bud mass per shoot (F). Error bars denote 95 % CI. Different letters indicate significant differences among crown locations (within each month), and different numbers indicate significant differences across sampling months (within each location). LI, lower-crown, interior-facing branches; LE, lower-crown, edge-facing branches; U, upper crown.

The characteristics of current-year shoots differed significantly across crown locations, and with the exception of total leaf area, depended also on sampling month (Table 3). Pair-wise comparisons showed that LI shoots had significantly smaller stem CSA than both LE or U shoots throughout the study period (Fig. 2A). The shoots from LI branches were also shorter than LE or U shoots, although the difference between LI and LE shoots was non-significant in August (Fig. 2B). Nevertheless, LI shoots consisted of significantly fewer nodes than both LE and U shoots throughout the study (Fig. 2C), and LI shoots also tended to support a smaller total leaf area (Fig. 2D).

**Table 3.** The effects of crown location and sampling month on shoot characteristics. Continuous variables (log_10_-transformed) were analysed using mixed-effects ANOVA and counts by mixed-effects Poisson regression (nested random effects: sample tree and branch).

Variable	Fixed effect			
	Crown location		Sampling month	
CSA of stem	*F*(2, 36) = 63.4	*P* < 0.001	*F*(2, 36) = 9.5	*P* = 0.001
Shoot length	*P*(2, 36) = 40.8	*P* < 0.001	*F*(2, 36) = 3.2	*P* = 0.05
Number of nodes	χ^2^(2) = 100.1	*P* < 0.001	χ^2^(2) = 15.4	*P* < 0.001
Total leaf area	*F*(2, 22) = 9.4	*P* = 0.001	*F*(1, 22) = 1.2	*P* = 0.3
Number of buds	χ^2^(2) = 132.5	*P* < 0.001	χ^2^(2) = 10.9	*P* = 0.005
Total bud mass	*F*(2, 36) = 68.0	*P* < 0.001	*F*(2, 36) = 157.7	*P* < 0.001

In contrast, the differences between LE and U shoots were mostly non-significant (Fig. 2A–D). In July, shoots had almost the same stem CSA and the number of nodes in both locations, but unlike lower-crown shoots, U shoots continued growing in late summer, so that significant increases were detected for both variables (Fig. 2A and C). Consequently, U shoots sampled in October had significantly greater stem CSA than LE shoots, although the number of nodes was still deemed similar by pair-wise comparisons.

Besides differences in other shoot characteristics, LI shoots also produced significantly fewer buds than LE or U shoots, while bud number was similar in the two latter locations throughout the study (Fig. 2E). Although sampling month had a significant effect on the number of buds (Table 3), pair-wise comparisons could not detect any differences in bud number in relation to sampling date (Fig. 2E). However, depending on the location, total bud mass per shoot increased 5–8 times during the study period. Total bud mass increased significantly in all three locations between July and August, and tended to further increase between August and October (Fig. 2F). Nevertheless, total bud mass in LI shoots remained significantly lower than elsewhere throughout the study, whereas the differences between LE and U shoots were non-significant.

### Leaf–stem allometry

**Figure 3.**
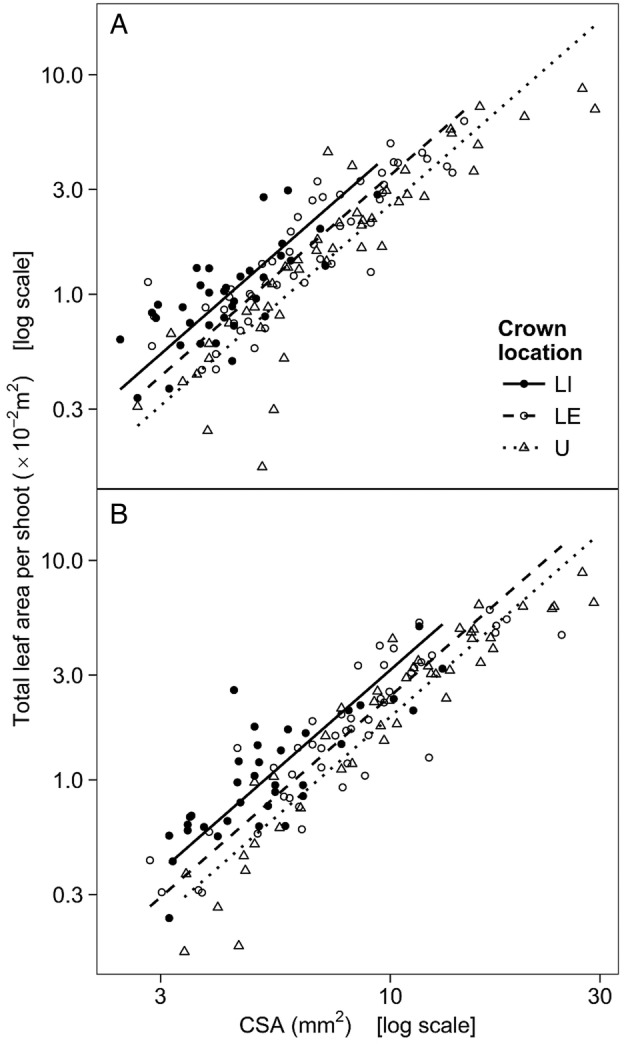
The allometry between the total leaf area per shoot and CSA of stem in mid-July (A) and late August (B). The lines represent SMA regressions, fitted with a common slope (1.75) across crown locations and sampling months. Note the log_10_-transformed axes. LI, lower-crown, interior-facing branches; LE, lower-crown, edge-facing branches; U, upper crown.

Total leaf area was highly correlated to stem CSA in all six groups (Table 4). The relationship between the two variables scaled similarly regardless of crown location or sampling month; however, the intercepts were non-homogeneous (Table 4). In general, upper-crown shoots supported a smaller total leaf area at a given stem CSA than lower-crown shoots, and the total leaf area per given stem CSA was reduced between July and August. Pair-wise comparisons revealed that the intercepts for U shoots differed significantly from LI and LE shoots both in July and in August, and intercepts also differed between sampling months within each location (Table 4, Fig. 3A and B).

**Table 4.** The allometry between total leaf area per shoot and the CSA of stem. Leaf–stem relationships were fitted using SMA estimation. As the relationships scaled similarly across each combination of crown location and sampling month (LR(5) = 1.74, *P* = 0.9), they were fitted with a common slope (presented with 95 % CI) across all groups. However, the intercepts were non-homogeneous (*W*(5) = 97.7, *P* < 0.001), so the global test was followed by pair-wise comparisons. Different letters denote significantly different intercepts among crown locations (within each month), and different numbers denote significant differences between sampling months (within each location). Correlation coefficients (*r*) are significant at *P* < 0.001. LI, lower-crown, interior-facing branches; LE, lower-crown, edge-facing branches; U, upper crown.

Sampling month		Crown location		
		LI	LE	U
	Common slope	1.75 (1.65 … 1.86)		
July				
	Intercept	−1.11 *a1*	−1.21 *a1*	−1.34 *b1*
	*r*	0.733	0.878	0.889
August				
	Intercept	−1.25 *a2*	−1.37 *a2*	−1.47 *b2*
	*r*	0.815	0.890	0.940

### Bud–stem allometry

**Figure 4.**
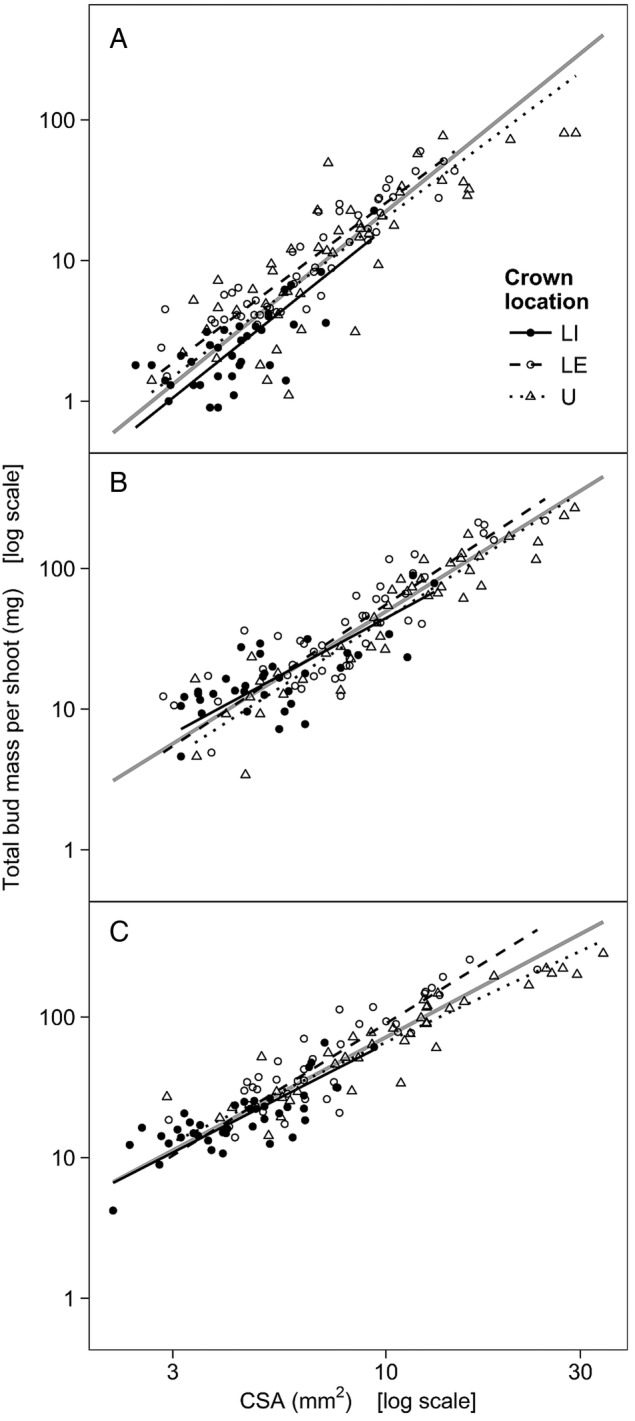
The allometry between total bud mass per shoot and the CSA of stem in mid-July (A), late August (B) and late October (C). The lines represent SMA regressions (grey lines represent fits for data that were pooled across crown locations). Note the log_10_-transformed axes.

Total bud mass per shoot and stem CSA were also highly correlated (Table 5). Testing for a common slope indicated that the scaling relationship differed across the nine groups (Table 5). Pair-wise comparisons revealed that slopes were similar among crown locations within each month. However, slopes tended to vary across sampling months: although significant differences were detected only for U shoots, slopes followed a similar, decreasing trend in all three crown locations. Thus, the data were pooled across crown locations, and the slopes fitted for each sampling month differed significantly from one another (Table 5, Fig. 4). Although the slopes remained positive (i.e. shoots with greater stem CSA had greater total bud mass in each sampling month), they gradually decreased towards the end of the study period (i.e. total bud mass increased at a faster rate with increasing stem CSA in shoots that were sampled earlier in the season).

**Table 5.** The allometry between total bud mass per shoot and the CSA of stem. Bud–stem relationships were fitted using SMA estimation. Slopes are presented with 95 % CI. As the slopes fitted for each combination of crown location and sampling month were significantly different (LR(8) = 40.15, *P* < 0.001), the global test was followed by pair-wise comparisons. Different letters denote significant differences among crown locations (within each month), and different numbers denote significant differences among sampling months (within each location). Bud–stem relationships were also fitted on data pooled across crown locations within each month, and significant differences among sampling months are denoted by different numbers. Correlation coefficients (*r*) are significant at *P* < 0.001. LI, lower-crown, interior-facing branches; LE, lower-crown, edge-facing branches; U, upper crown.

Sampling month		Crown location			Pooled
		LI	LE	U	
July					
	Slope	2.32 (1.83 … 2.93) *a1*	2.18 (1.94 … 2.44) *a1*	2.16 (1.84 … 2.54) *a1*	2.35 (2.16 … 2.55) *1*
	Intercept	−4.08	−3.77	−3.86	−4.00
	*r*	0.735	0.919	0.839	0.877
August					
	Slope	1.57 (1.23 … 2.01) *a1*	1.92 (1.65 … 2.24) *a1*	1.88 (1.68 … 2.11) *a1*	1.80 (1.66 … 1.95) *2*
	Intercept	−2.92	−3.18	−3.24	−3.10
	*r*	0.708	0.861	0.930	0.890
October					
	Slope	1.48 (1.21 … 1.81) *a1*	1.79 (1.54 … 2.09) *a1*	1.36 (1.19 … 1.54) *a2*	1.54 (1.43 … 1.66) *3*
	Intercept	−2.67	−2.84	−2.54	−2.68
	*r*	0.779	0.884	0.921	0.915

**Figure 5.**
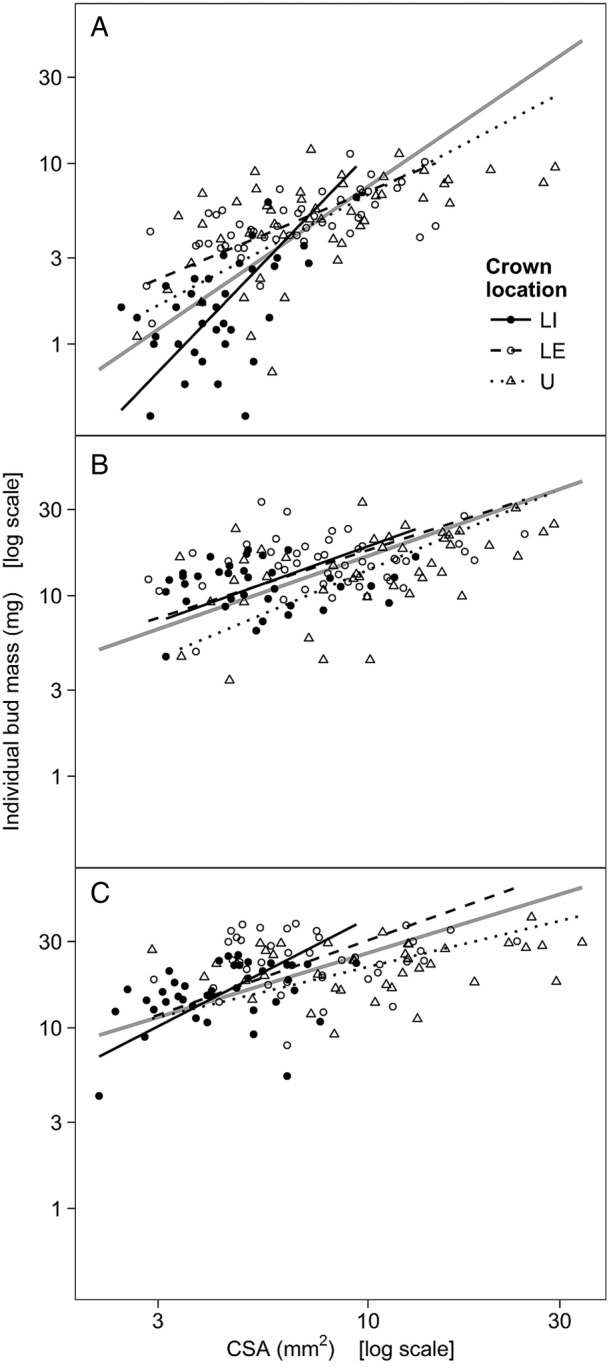
The allometry between individual bud mass and CSA of stem in mid-July (A), late August (B) and late October (C). The lines represent SMA regressions (grey lines represent fits for data that were pooled across crown locations). Note the log_10_-transformed axes.

Testing for a common slope revealed that the allometry of individual bud mass and stem CSA scaled differently across the nine groups (Table 6). However, the scaling slopes did not follow any apparent pattern across crown locations or sampling months, and the correlations between individual bud mass and stem CSA were weak and mostly non-significant, except in July (Table 6). Pooled across crown locations, the correlations between individual bud mass and stem CSA were significant in each sampling month, and the relationships were positive (i.e. shoots with greater stem CSA had larger buds; Table 6). The slope fitted on pooled data was significantly steeper in July than in August or October, resulting mainly from the fact that the slope fitted for LI shoots in July was particularly steep (Table 6, Fig. 5).

**Table 6.** The allometry between individual bud mass and CSA of stem. Bud–stem relationships were fitted using SMA estimation. Slopes are presented with 95 % CI. As the slopes fitted for each combination of crown location and sampling month were significantly different (LR(8) = 52.4, *P* < 0.001), the global test was followed by pair-wise comparisons. Different letters denote significant differences among crown locations (within each month), and numbers denote significant differences among sampling months (within each crown location). Bud–stem relationships were also fitted on data pooled across crown locations within each month, and significant differences among sampling months are denoted by different numbers. Significance levels for correlation coefficients (*r*): ***P < 0.001; **P < 0.01; *P < 0.05; ns *P* > 0.05. LI, lower-crown, interior-facing branches; LE, lower-crown, edge-facing branches; U, upper crown.

Sampling month		Crown location			Pooled
		LI	LE	U	
July					
	Slope	2.31 (1.73 … 3.08) *a1*	0.93 (0.75 … 1.16) *b1*	1.17 (0.92 … 1.49) *b1*	1.51 (1.33 … 1.72) *1*
	Intercept	−4.25	−3.08	−3.34	−3.64
	*r*	0.538***	0.656***	0.565***	0.657***
August					
	Slope	0.80 (0.56 … 1.13) *a2*	0.72 (0.54 … 0.96) *a1*	0.93 (0.71 … 1.23) *a12*	0.77 (0.66 … 0.91) *2*
	Intercept	−2.52	−2.47	−2.79	−2.55
	*r*	0.083 ns	0.262 ns	0.477**	0.389***
October					
	Slope	1.14 (0.84 … 1.55) *a2*	0.79 (0.57 … 1.09) *ab1*	0.53 (0.39 … 0.73) *b2*	0.68 (0.58 … 0.80) *2*
	Intercept	−2.54	−2.31	−2.20	−2.27
	*r*	0.359*	0.114 ns	0.214 ns	0.380***

## Discussion

In the current study, we investigated crown development in young black alder trees. Identifying the factors that regulate bud and shoot growth provides an insight into the mechanisms determining plant architecture, which is a key component of plant growth modelling, a method used widely in the fields of environmental sciences, agriculture and forestry (Fourcaud *et al*. 2008).

We found that individual bud mass was determined by local light conditions rather than by branch height, as upper- and lower-crown buds differed little in the absence of a steep light gradient. Similarly, it has been shown that artificially shading the top branches induces bud characteristics similar to those found further drown the crown (Jones and Harper 1987*a*, *b*; Kimura *et al*. 1998; Uemura *et al*. 2000). As defoliation leads to a decrease in bud size (Marcelis-van Acker 1994), it is likely that light availability promotes bud development through assimilate supply. However, light also acts as a developmental cue: both the intensity and quality of light affect bud outgrowth in rose, and the light signal is perceived by buds, not by the shoot (Girault *et al*. 2008).

Nevertheless, in the current study, the average number of leaf primordia per bud varied little across the crown. Similarly, Gordon *et al*. (2006) and Taugourdeau and Sabatier (2010) found that light availability had little effect on the number of preformed leaves in peach and walnut, respectively. In contrast, Kimura *et al*. (1998) report that shoot morphology in beech is largely determined by previous-year light conditions, presumably via preformation in the bud. In general, larger buds contain a greater number of preformed organs (e.g. Remphrey and Davidson 1994; Cochard *et al*. 2005; Lauri *et al*. 2008). However, the current study found that although higher light availability increased individual bud mass, the number of leaf primordia per bud was unaffected, suggesting that the initiation and growth of leaf primordia were decoupled. Likewise, Henry *et al*. (2011) observed that bud elongation in rose was highly diminished in response to sugar starvation during bud outgrowth, whereas organogenesis remained constant. According to Gordon *et al*. (2006), preformation in peach is little affected by a range of both exogenous and endogenous factors (e.g. light availability, drought, tree carbohydrate status), and plasticity in crown architecture is mostly achieved via neoformation, by initiating new organs that extend during the same growing season. Thus, the limited impact of light availability on organ initiation within the bud may represent a more widespread strategy among species relying on neoformed growth (Guédon *et al*. 2006).

Although leaf–stem allometry was affected by branch height, shoot characteristics corresponded mainly with local light availability, consistent with previous studies (Kimura *et al*. 1998; Kull and Tulva 2002; Osada *et al*. 2004). Upper-crown shoots and well-lit lower-crown shoots had greater stem CSA, they were longer and consisted of a greater number of nodes than the shaded lower-crown shoots. These shoots also produced more leaves and buds and consequently supported a greater total leaf area and total bud mass per shoot. Most likely, light availability stimulated the different aspects of shoot growth via assimilate supply. Unlike both lower-crown locations, however, upper-crown shoots exhibited late-summer growth. Similarly, a greater extent of neoformation in the upper versus lower crown was observed by Davidson and Remphrey (1994). In the current study, light availability was slightly better in the upper crown than in the well-lit lower branches, and it remains unknown whether comparable light conditions would trigger late-summer growth in the lower crown. Goulet *et al*. (2000) observed that, depending on species, differences in shoot growth either correspond with local light availability, or the effect of light availability is modulated by branch height, so that only upper-crown shoots are capable of utilizing the more favourable conditions.

The scaling slopes of leaf–stem allometry remain constant in a wide range of circumstances, whereas the intercepts vary, reflecting an adjustment to hydraulic or mechanical stress (McDowell *et al*. 2002; Preston and Ackerly 2003; Westoby and Wright 2003; Bucci *et al*. 2005; Sun *et al*. 2006; Normand *et al*. 2008). Similarly, upper-crown shoots supported a smaller total leaf area per given stem CSA in the current study, most likely compensating for the hydraulic limitation imposed by the longer resource transport pathways (c.f. McDowell *et al*. 2002). In addition, total leaf area per given stem CSA was reduced in all three crown locations between mid-July and late August. The change was driven by leaf shedding rather than radial growth, as the latter was restricted to upper-crown shoots. In black alder, leaf shedding commonly starts in the inner parts of the crown as early as July, while new leaves are continuously produced in the periphery of the crown (Eschenbach and Kappen 1996).

Unlike leaf–stem relationships, allometric studies rarely involve buds. Nevertheless, climatic conditions, namely temperature and rainfall, are known to modulate bud–stem allometry, so that steeper scaling slopes are found in milder climate (Alla *et al*. 2011). The mechanisms linking bud growth and stems have remained unclear. Cochard *et al*. (2005) hypothesize that enhanced bud development in shoots with greater xylem area is associated with improved hydraulic supply. Supporting this notion, meristem vigour decreases as trees mature, manifested in the production of small, low-vigour shoots. According to grafting studies, reduced shoot vigour is related to increasing tree height and possibly involves hydraulic constraints (Bond *et al*. 2007). Nevertheless, the present study found that branch height as well as local light conditions had little effect on the allometry of total bud mass and the CSA of stem. Homogeneous scaling slopes may reflect a limited height difference between upper and lower branches: although the sampling locations were sufficiently far apart to influence leaf–stem allometry, developing buds are far less susceptible to water shortage than leaves (Barigah *et al*. 2013). Alternatively, the mechanisms regulating bud–stem allometry may act locally.

Each new generation of buds is formed in mid-summer, followed by a period of rapid bud development in late summer and fall (Eschrich *et al*. 1989; Alla *et al*. 2013*a*). Similarly, both individual and total bud mass increased multiple times during the current study, but allometries revealed that total bud mass increased disproportionally less in thicker shoots regardless of crown location. As late-summer growth was restricted to the upper crown, the CSA of stem and bud number remained the same in lower-crown shoots throughout the study, so changes in the allometry of total bud mass and stem CSA were mainly driven by the growth of individual buds. The allometry of individual bud mass and stem CSA revealed that larger shoots carried larger buds, but slopes also tended to decrease across sampling months. However, unlike the clear pattern for total bud mass, the difference mainly stems from a particularly steep slope fitted for shaded lower-crown shoots in July. In late summer, shoot and bud abscission were relatively common in shaded parts of the crown, and the fact that poorly-developed shoots were eliminated later in the season may explain the anomalous slope. Unlike total bud mass, however, the correlations between individual bud mass and stem CSA were relatively strong in July, but mostly weak on later sampling dates. Gill (1971) found that initially, bud size varies little along a parent shoot, but inequalities become evident in late summer, so increasing differences may also be responsible for the lower correlation of individual bud mass and the CSA of stem in the current study.

Within a branching zone, well-developed buds are characterized by higher xylem hydraulic conductance in spring (Lauri *et al*. 2008), so the differences in bud size and contents may be linked to vascular differentiation. A possible mechanism connecting the two processes involves auxin signalling (Sachs *et al*. 1993). Auxins are synthesized in young, developing leaves, but low auxin levels are necessary for maintaining leaf initiation and growth (Domagalska and Leyser 2011), so for bud growth to continue, excess auxin needs to be exported. Via positive feedback, auxin transport is canalized into specialized files of cells, which may later differentiate into vascular strands (Berleth and Mattsson 2000). As auxin sources compete for a common transport pathway to the root (Domagalska and Leyser 2011), competition among adjacent buds may also be responsible for the changes in the allometry of total bud mass and stem CSA that were observed in the current study. However, other explanations are possible as well: for example, buds supported by thicker shoots may have reached their final size earlier.

## Conclusions

In young black alder trees, crown development is influenced by local environment as well as endogenous processes. Bud size in the upper versus lower crown depended mainly on light availability; however, the number of preformed leaf primordia per bud varied little across the crown. Consequently, the plasticity in shoot size and bud production, driven by local light availability, is mostly achieved by neoformation. Unlike shoot characteristics, leaf–stem allometry depended on branch height, most likely reflecting hydraulic stress due to longer resource transport pathways. In contrast, bud–stem allometry was unaffected by branch height, but scaling slopes varied throughout the sampling period. Varying scaling was driven by bud growth and suggests that bud–stem allometry is regulated locally rather than at the crown level.

## Sources of Funding

Our work was funded by the Estonian Science Foundation (grant No. 9186), the European Regional Development Fund (Centre of Excellence in Environmental Adaptation; project No. F11100PKTF) and the Estonian Ministry of Education and Research (institutional research funding IUT 34-9).

## Contributions by the Authors

M.K. developed the sampling protocol, collected and analysed the data, and wrote the paper. A.S. conceived the study and provided feedback during manuscript writing.

## Conflict of Interest Statement

None declared.
